# Changes in foveal avascular zone area and retinal vein diameter in patients with retinal vein occlusion detected by fundus fluorescein angiography

**DOI:** 10.3389/fmed.2023.1267492

**Published:** 2023-11-10

**Authors:** Dingying Liao, Zixia Zhou, Fei Wang, Bin Zhang, Yanfen Wang, Yuping Zheng, Jinying Li

**Affiliations:** ^1^Department of Ophthalmology, Shenzhen Hospital, Peking University, Shenzhen, China; ^2^Department of Ophthalmology, Second Affiliated Hospital, Xi’an Jiaotong University, Xi’an, China

**Keywords:** retinal vein occlusion, fundus fluorescein angiography, intravitreal injection, foveal avascular zone, vessel diameter, best corrected visual acuity

## Abstract

**Purpose:**

To investigate changes in foveal avascular area (FAZ) and retinal vein diameter in patients with retinal vein occlusion (RVO) after intravitreal ranibizumab, and to analyze the correlation between ranibizumab therapy and visual gain.

**Methods:**

This retrospective study enrolled 95 eyes of 95 patients who had accepted three consecutive monthly ranibizumab injections, including 50 branch RVOs (BRVOs) and 45 central RVOs (CRVOs). BRVOs were divided into ischemia group (*n* = 32) and non-ischemia group (*n* = 18), and CRVOs also had ischemia group (*n* = 28) and non-ischemia group (*n* = 17). Comprehensive ophthalmic examinations were performed before the first injection and after 6, 12, and 24 months. The FAZ was manually circumscribed on early-phase images of fundus fluorescein angiography. Retinal vein diameters were measured on fundus photographs.

**Results:**

After three injections, the FAZ area was significantly enlarged firstly and then reduced in all ischemic RVOs and the non-ischemic BRVOs (*p* < 0.05), while the retinal vein diameter was significantly reduced firstly and then increased in all groups except for unobstructed branch veins of non-ischemic BRVOs (*p* < 0.05). The correlation between the FAZ area and best corrected visual acuity was statistically significant in all CRVOs (non-ischemic, *r* = 0.372; ischemic, *r* = 0.286; *p* < 0.01) and ischemic BRVOs (*r* = 0.180, *p* < 0.05). Spearman’s correlation analysis revealed that the retinal vein diameter was significantly correlated to the larger FAZ area in obstructed branch veins of ischemic BRVOs (*r* = −0.31, *p* < 0.01), inferior temporal branch veins of non-ischemic CRVOs (*r* = −0.461, *p* < 0.01) and ischemia CRVO groups (superior temporal branch vein, *r* = −0.226, *p* < 0.05; inferior temporal branch vein, *r* = −0.259, *p* < 0.01).

**Conclusion:**

After three consecutive monthly ranibizumab injections, the FAZ area was enlarged and retinal vein diameter reduced with gradual recovery to near baseline from 12 months. These results suggest that ranibizumab therapy can worsen macular ischemia and prevent visual gain in the short term. It has important significance for the treatment and prognosis of RVO, although the natural course of RVO may also affect ischemia and visual gain.

## 1. Introduction

Retinal vein occlusion (RVO) is a prevalent retinal vascular disorder, ranking second after diabetic retinopathy (1). Its incidence rises with age, affecting predominantly elderly individuals, with an estimated global prevalence of over 16 million people (2). RVO encompasses two main types: branch retinal vein occlusion (BRVO) and central retinal vein occlusion (CRVO). Common risk factors associated with RVO include diabetes, hypertension, dyslipidemia, and smoking. The condition manifests through retinal ischemia, vascular tortuosity, retinal hemorrhage, and macular edema due to obstruction of retinal veins at arteriovenous crossings (3).

Fundus fluorescein angiography (FFA) has long been established as the gold standard diagnostic tool for evaluating retinal vascular diseases, including RVO (4). This technique involves the intravenous injection of fluorescein dye, followed by capturing sequential images of the dye’s circulation within the retinal vessels using a specialized camera (5). By analyzing the dynamics of the dye’s distribution, FFA provides valuable information about the perfusion status, presence of vascular abnormalities, and alterations in the foveal avascular zone (FAZ) associated with RVO (6, 7).

The evaluation of RVO using FFA enables clinicians to visualize and assess the extent of retinal vascular involvement (8). The presence of venous obstruction, areas of non-perfusion, capillary leakage, and neovascularization can be detected, aiding in the accurate diagnosis and classification of RVO (9). Additionally, FFA allows for the precise assessment of FAZ alterations, which play a crucial role in determining visual outcomes in patients with retinal vascular diseases (10).

Through FFA, clinicians can analyze the FAZ area, which represents the central region of the retina devoid of blood vessels. Changes in FAZ area, such as enlargement or irregularity, have been associated with the severity and prognosis of RVO (11–13). These alterations can reflect the extent of retinal ischemia and provide insights into the functional impairment experienced by patients.

The combination of FFA with advanced image analysis techniques allows for a more comprehensive evaluation of RVO. Image analysis algorithms can provide quantitative measurements and objective assessments of FAZ area and other vascular parameters, reducing subjectivity and potential diagnostic errors associated with manual interpretation (14). This integration enhances the accuracy and reliability of RVO diagnosis and monitoring.

In this study, we aim to evaluate FAZ area and retinal vein diameter changes in eyes with RVO after ranibizumab therapy. We will utilize the gold standard FFA technique to assess the perfusion status, vascular abnormalities, and alterations in the FAZ. Incorporating advanced image analysis algorithms will enable us to obtain objective and quantitative measurements of these parameters. By investigating the correlations between FAZ area, retinal vein diameter, and best-corrected visual acuity (BCVA) following intravitreal ranibizumab treatment, we can gain a better understanding of the effects of anti-VEGF therapy on retinal vascular and structural parameters and their relationship with visual outcomes in RVO patients.

## 2. Materials and methods

### 2.1. Study population and design

In this retrospective, observational, and consecutive series study, 235 patients with RVO who visited the Ophthalmology Department of the Second Affiliated Hospital of Xi’an Jiaotong University (Shaanxi, China) were enrolled from August 2020 to September 2022. This study ultimately included 50 eyes of 50 patients with BRVO and 45 eyes of 45 patients with CRVO that were treated with ranibizumab (Lucentis; Genentech, San Francisco, CA, United States) by three consecutive intravitreal injection. These patients have received 6–8 injections (3 consecutive and 3–5 PRN treatments) within 24 months, with the decision for PRN treatments based on the degree of macular edema observed via OCT. In CRVO patients, an FFA result indicating a total area of non-perfusion in retinal capillaries greater than 10 DA (disc area) is classified as ischemic, while less than or equal to 10 DA is classified as non-ischemic. For BRVO patients, an FFA result demonstrating a total area of non-perfusion in retinal capillaries greater than 5 DA is considered ischemic, whereas less than or equal to 5 DA is categorized as non-ischemic. The 50 BRVOs were divided into an ischemia group (*n* = 32) and a non-ischemia group (*n* = 18). The 45 CRVOs were also divided into an ischemia group (*n* = 28) and a non-ischemia group (*n* = 17). These 95 patients accepted injections monthly for three consecutive months. All patients signed an informed consent form. This study procedure adhered to the tenets of the Declaration of Helsinki.

Demographic data and clinical records of systemic diseases, medical and ophthalmic histories were obtained from all patients. Each patient underwent comprehensive ophthalmic examinations the day before and the day after treatment. FFA was performed for each patient before 6, 12, and 24 months after the first intravitreal injection. The inclusion criteria were: (1) treatment-naive RVO patients; and (2) BCVA of 20/50 or worse if there were no other existing ocular diseases, including media opacity, uveitis, diabetic retinopathy, retinal arterial occlusion, glaucoma, and age-related macular degeneration. Patients with high myopia (over 8D), high astigmatism (over 3D), epiretinal membrane, or a history of pars plana vitrectomy were excluded. Eyes with poor-quality images on FFA due to eye movement were also excluded.

### 2.2. Ranibizumab injections

Intravitreal ranibizumab injections were administered in the operating room under strictly sterile conditions. Each patient was given an intravitreal injection of ranibizumab (0.05 mL), which was administered with a 30G needle inserted at 4 mm (phakic eyes) or 3.5 mm (pseudophakic eyes) from the limbus. The light perception of the eye was confirmed immediately after injection. In the following 3 days, the slit lamp examination was performed to check for any possible intraocular inflammation.

### 2.3. Fundus fluorescein angiography and measurement of the FAZ area

**Figure 1:**
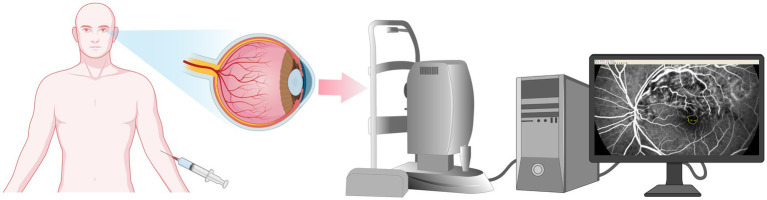
Fundus fluorescein angiography (FFA) platform.

For the measurement of the FAZ area, FFA was performed with a scanning laser device known as the Heidelberg Spectralis HRA (Heidelberg Retina Angiograph II; Heidelberg Engineering, Heidelberg, Germany) (Figure 1). RVO was defined by delayed venous filling in the region of retinal nonperfusion and obstructed veins, which may be caused by microvascular circulation abnormalities. The boundaries of the FAZ were manually delineated by two independent observers who were masked to the BCVA, and the area of FAZ was automatically measured by the software of the device in early-phase images after it was delineated (15).

### 2.4. Other ophthalmic parameters and their measurement

Fundus images were captured with a fundus camera (Topcon Imagenet, Tokyo, Japan). Two independent observers manually measured the vertical diameters of the superior and inferior temporal retinal veins using Adobe Photoshop CC (Adobe Systems, Inc. San Jose, CA, United States). The BCVA of each patient was measured at a distance of 4 m (or 1 m if necessary) following the guidance of the Early Treatment Diabetic Retinopathy Study (ETDRS) charts.

### 2.5. Statistical analyses

For statistical analysis, the BCVA was measured with ETDRS charts and converted to the logarithm of the minimal angle of resolution (LogMAR) scale. Comparisons between the BCVA before and after treatment were calculated using the nonparametric Wilcoxon signed rank tests. The bivariate relationships were assessed with the Spearman’s correlational coefficient. All statistical analyses were performed using IBM SPSS Statistics version 21.0 (SPSS Inc., Chicago, IL, United States). A *p* value of <0.05 was considered statistically significant.

## 3. Results

### 3.1. Clinical characteristics of patients

A total of 235 patients with RVO were initially enrolled in this study, and 140 were excluded according to criteria. Therefore, 95 eyes of 95 subjects (50 BRVO patients and 45 CRVO patients) who received three consecutive and PRN ranibizumab injections met the criteria for the present study.

The clinical characteristics and outcomes of all patients are summarized in Table 1. The mean age of the patients with BRVO was 58.92 years (range, 35–82 years), and the mean age of the patients with CRVO was 63.20 years (range, 39–88 years). The mean BCVA was improved from 0.47 ± 0.21 before treatment to 0.45 ± 0.25 after follow-up in the BRVO group, and from 0.64 ± 0.23 before treatment to 0.61 ± 0.32 after follow-up in the CRVO group.

**Table 1:** Baseline clinical characteristics and outcome of patients with retinal vein occlusion.

	BRVO (*n* = 50)	CRVO (*n* = 45)
Age (years)	58.92 ± 11.19	63.20 ± 12.18
Sex (*n*, F/M)	18/32	20/25
Period from onset to 1st injection treatment (weeks)	5.08 ± 3.80	3.56 ± 2.69
**Systemic diseases**		
Hypertension (*n*, %)	31, 62%	33, 73%
Diabetics mellitus (*n*, %)	26, 52%	28, 62%
Hypercholesteremia (*n*, %)	20, 40%	27, 60%
**BCVA (logMAR)**		
Before treatment	0.47 ± 0.21	0.64 ± 0.23
After follow-up	0.45 ± 0.25	0.61 ± 0.32
**Foveal thickness (um)**		
Before treatment	409.38 ± 182.48	410.17 ± 148.13
After follow-up	201.63 ± 86.57	343.83 ± 127.45

N, number of eyes; %, rate of eyes; BRVO, branch retinal vein occlusion; CRVO, central retinal vein occlusion; BCVA, best correct visual acuity; MAR, minimum angle of resolution; F, female; M, male.

### 3.2. The trend of changes in the FAZ area from baseline to follow-up

The mean size of the FAZ changed from 0.44 ± 0.30 mm^2^ to 0.48 ± 0.31 mm^2^ in non-ischemia BRVO patients and from 0.61 ± 0.22 mm^2^ to 0.67 ± 0.31 mm^2^ in ischemia BRVO patients. For CRVO patients, the mean FAZ area changed from 0.60 ± 0.27 mm^2^ to 0.61 ± 0.38 mm^2^ in the non-ischemia group and from 0.69 ± 0.21 mm^2^ to 0.76 ± 0.25 mm^2^ in the ischemia group.

**Figure 2:**
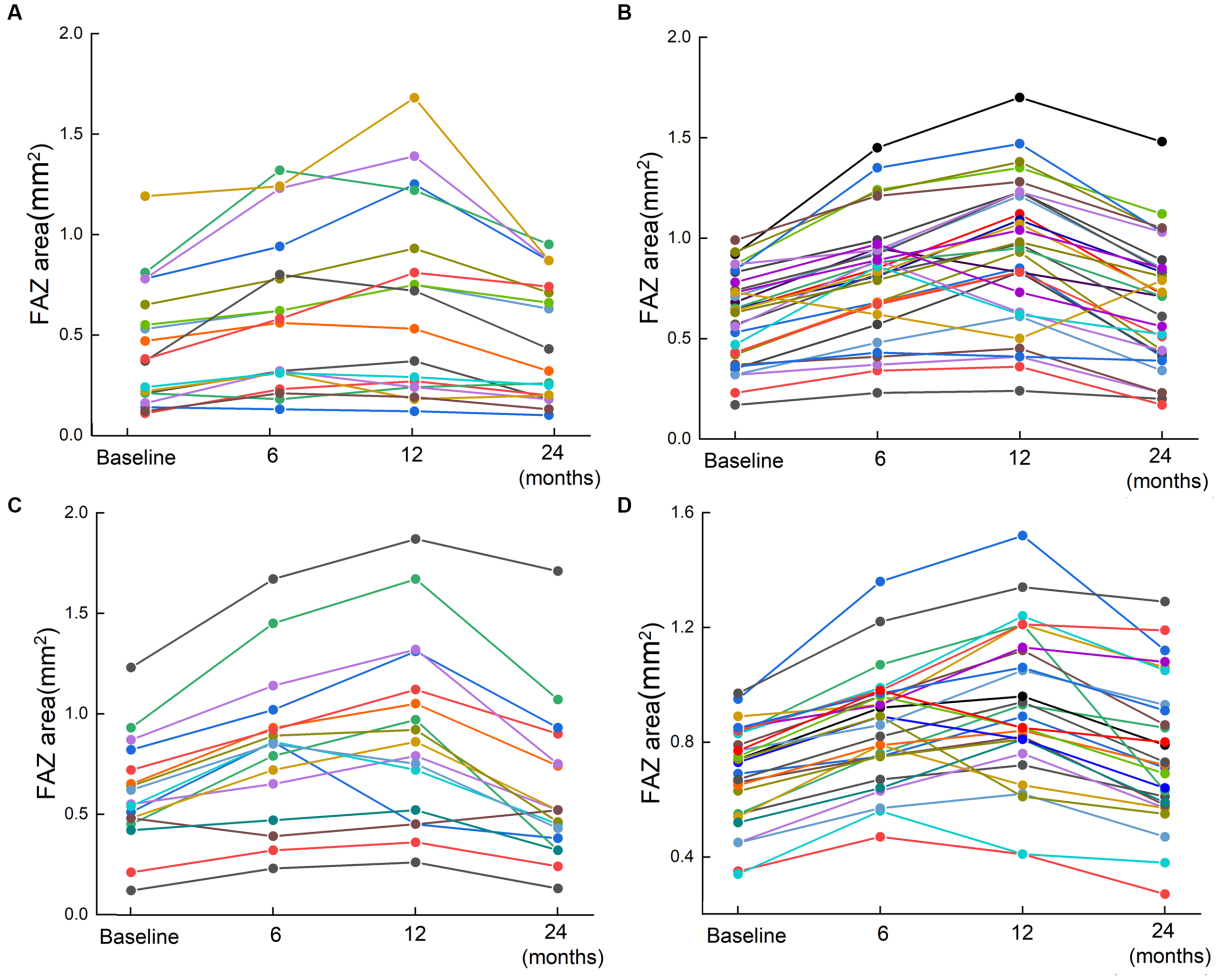
The changes of FAZ area for 95 patients based on FFA results. Scatter plots for the FAZ area in patients with RVO before the first injection and 6, 12, and 24 months after the first injection. **(A)** Non-ischemia BRVO; **(B)** Ischemia BRVO; **(C)** Non-ischemia CRVO; **(D)** Ischemia CRVO. Changes of the FAZ after treatment are represented by lines. Each line shows the trend of FAZ area changes for one patient. The four points indicated the FAZ area at baseline (before injection) and 6, 12, and 24 months after the first injection.

**Figure 3:**
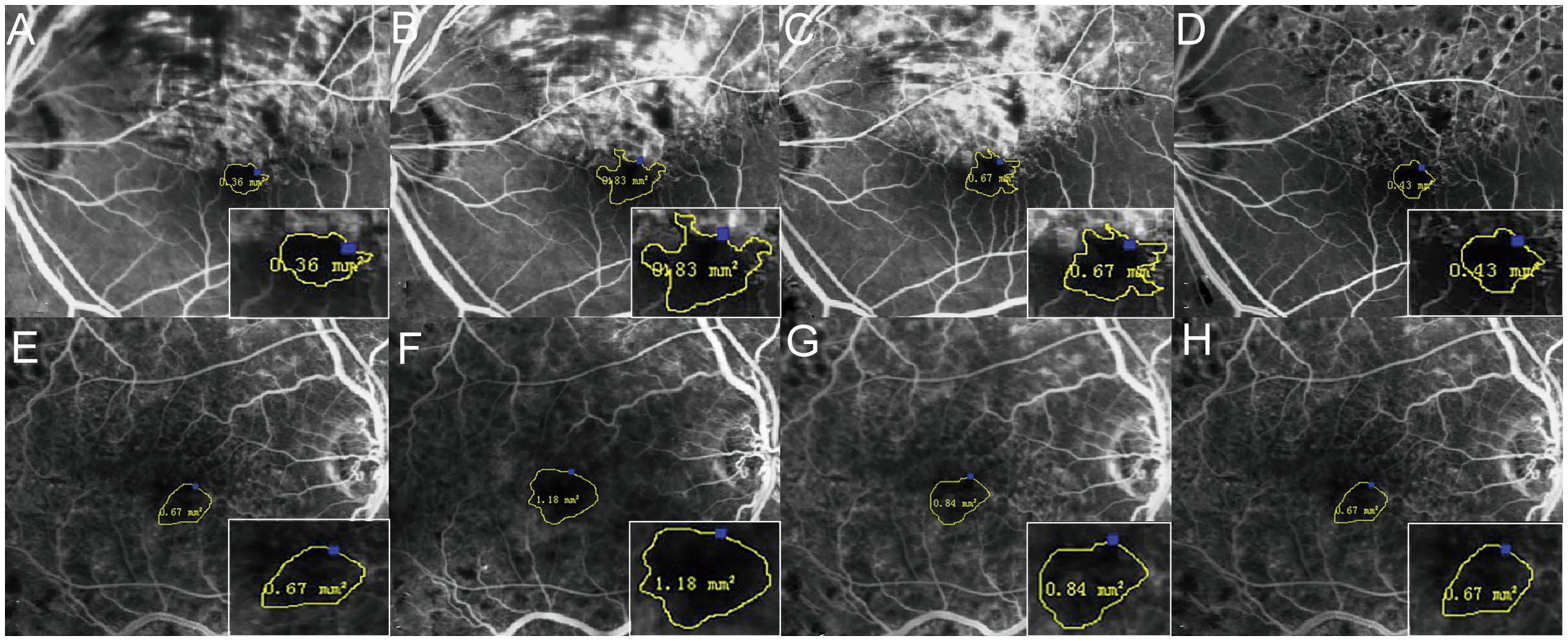
Changes of the FAZ area before and 6, 12, and 24 months after the first injection. The four times measurement of FAZ area for one of the BRVO patients **(A–D)** and one of the CRVO patients **(E–H)** by FFA. The FAZ area was enlarged until 12 months, and it then reduced gradually.

Most patients with ischemic and non-ischemic BRVO and CRVO had an obviously enlarged FAZ area after ranibizumab treatment, and a small number of patients had a reduced FAZ area, whereas only a few patients showed a stable FAZ area after three injections. The FAZ area was enlarged after ranibizumab treatment and persisted for a while, and then it reduced gradually. Compared with baseline, there was a significant increase in the size of the FAZ area at 6 and 12 months after the first injection. At the last FFA (24 months after the first injection), most cases showed an obvious decrease in the size of the FAZ, although it was still a little bigger than the pretreatment baseline value (Table 2; Figures 2, 3).

**Table 2:** The trend of changes in FAZ area and retinal vein diameter before treatment and follow-up.

Parameter	Baseline	6 months	12 months	24 months	*p*
**FAZ area(mm^2^)**					
BRVO					
Non-ischemia	0.44 ± 0.30	0.59 ± 0.39	0.66 ± 0.48	0.48 ± 0.31	0.256
Ischemia	0.61 ± 0.22	0.81 ± 0.29	0.92 ± 0.36	0.67 ± 0.31	0.000**
CRVO					
Non-ischemia	0.60 ± 0.27	0.83 ± 0.37	0.91 ± 0.45	0.61 ± 0.38	0.042*
Ischemia	0.69 ± 0.21	0.85 ± 0.20	0.92 ± 0.27	0.76 ± 0.25	0.001**
**Vessel diameter (×10^−2^ mm)**					
BRVO					
Non-ischemia					
Obstructed BV	20.89 ± 1.74	19.00 ± 1.76	18.81 ± 2.20	19.69 ± 2.57	0.018*
Unobstructed BV	16.54 ± 2.93	14.46 ± 3.23	14.75 ± 2.25	16.06 ± 1.89	0.057
Ischemia					
Obstructed BV	20.33 ± 2.25	18.00 ± 2.12	17.93 ± 1.91	18.71 ± 3.39	0.000**
Unobstructed BV	17.05 ± 3.15	14.53 ± 3.01	14.53 ± 2.84	15.80 ± 2.84	0.002**
CRVO					
Non-ischemia					
Superior-temporal BV	18.42 ± 1.56	15.94 ± 1.53	16.62 ± 1.76	17.14 ± 1.99	0.001**
Inferior-temporal BV	19.68 ± 1.40	16.7 ± 1.59	17.82 ± 1.69	18.72 ± 1.92	0.000**
Ischemia					
Superior-temporal BV	20.33 ± 1.99	17.45 ± 1.85	16.97 ± 2.19	19.12 ± 2.37	0.000**
Inferior-temporal BV	21.96 ± 1.95	19.32 ± 2.12	18.21 ± 1.31	19.82 ± 2.03	0.000**

FAZ, foveal avascular zone; BRVO, branch retinal vein occlusion; CRVO, central retinal vein occlusion; BV, branch vein. * *p* < 0.05, ** *p* < 0.01.

### 3.3. Changes of retinal vein diameter before treatment and follow-up

**Figure 4:**
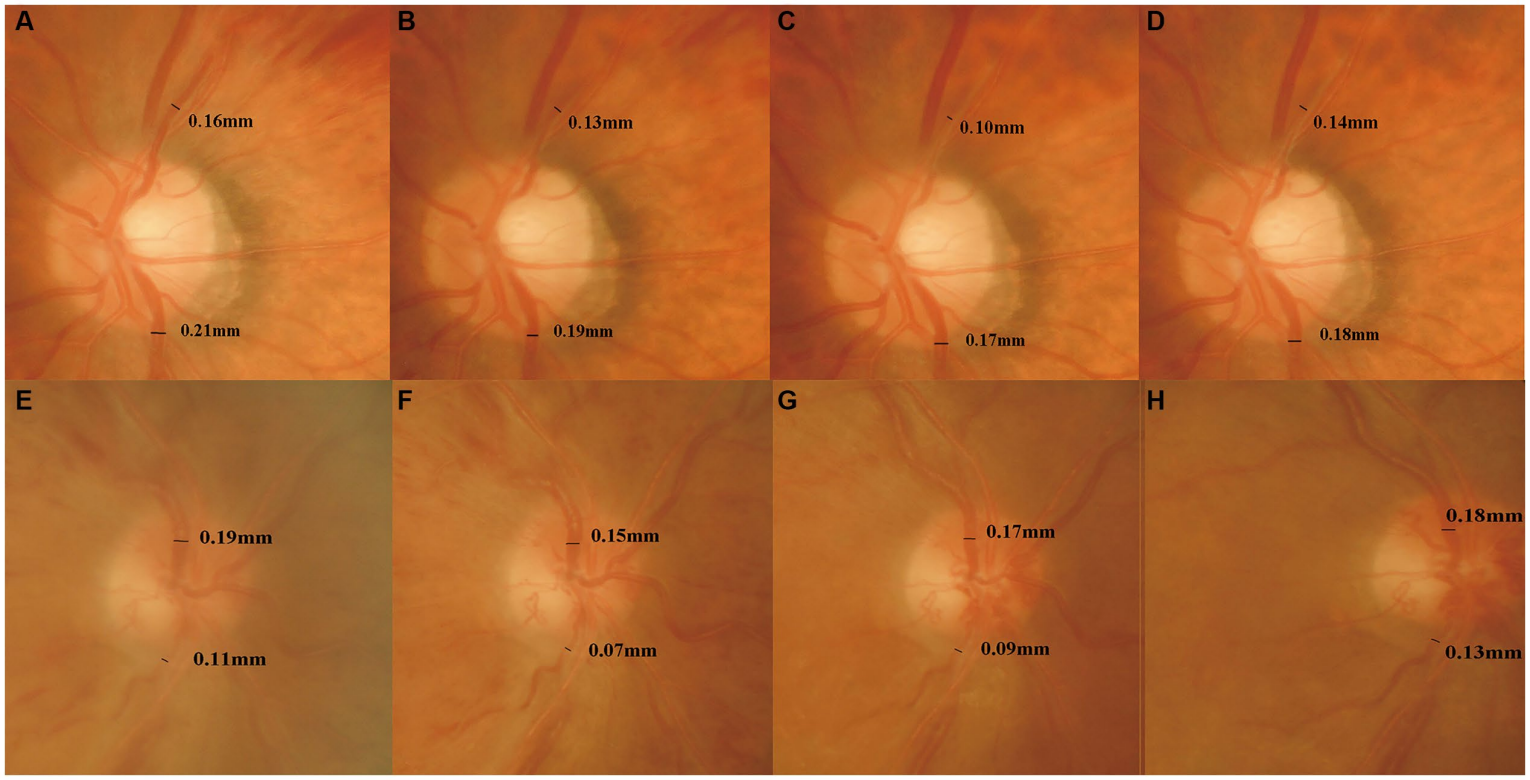
Changes of the retinal vein diameter before and 6, 12, and 24 months after the first injection. The four times measurement of retinal vein diameter for one of the BRVO patients **(A–D)** and one of the CRVO patients **(E–H)** in fundus photographs. The retinal vein diameter was decreased until 12 months, and then it increased gradually.

The retinal vein diameter data used in the present study were obtained by measuring the obstructed and unobstructed branch veins in BRVO patients and the superior and inferior temporal branch veins in CRVO patients. Except for the unobstructed branch veins of the non-ischemic BRVO group, the retinal vein diameters were significantly reduced after ranibizumab treatment and then recovered gradually (*p* < 0.05), and these tendencies were the same with the FAZ area (Table 2; Figure 4).

### 3.4. Changes correlations between FAZ area, retinal vein diameter, and BCVA

After ranibizumab treatment, LogMAR visual acuity significantly reduced along with FAZ area enlargement in eyes with CRVO groups (non-ischemic, *r* = 0.327, *p* < 0.001; ischemic, *r* = 0.286, *p* < 0.001) and in eyes with ischemic BRVO group (*r* = 0.180, *p* < 0.05), but not in eyes with non-ischemic BRVO (*r* = 0.200, *p* > 0.05) (Table 3).

**Table 3:** The correlation between FAZ area and BCVA (LogMAR).

Parameter	FAZ area	BCVA (LogMAR)	*r*	*p*
BRVO				
Non-ischemia	0.54 ± 0.38	0.43 ± 0.20	0.200	0.092
Ischemia	0.75 ± 0.32	0.54 ± 0.24	0.180	0.041*
CRVO				
Non-ischemia	0.74 ± 0.39	0.60 ± 0.29	0.327	0.007**
Ischemia	0.81 ± 0.25	0.72 ± 0.27	0.286	0.002**

BRVO, branch retinal vein occlusion; CRVO, central retinal vein occlusion; BCVA, best corrected visual acuity; FAZ, foveal avascular zone; BV, branch vein. * *p* < 0.05, ** *p* < 0.01.

There was a significantly negative correlation between the FAZ area and the diameter of the obstructed branch veins in ischemic BRVO group (*r* = −0.310, *p* < 0.01), whereas the non-ischemia BRVO groups and the unobstructed branch veins of ischemia BRVO group did not show such correlation (*p* > 0.05). The branch vein diameter of ischemic CRVO groups and the inferior temporal branch vein diameter of non-ischemic CRVO group had a significant negative association with the FAZ area (*p* < 0.05), but the superior temporal branch veins of the non-ischemia CRVOs did not show the same tendency (*p* > 0.05) (Table 4).

**Table 4:** The correlation between FAZ area and retinal vessel diameter.

Parameter	FAZ area (mm^2^)	Vessel diameter (×10^−2^ mm)	*r*	*p*
BRVO				
Non-ischemia	0.54 ± 0.38			
Obstructed BV		19.60 ± 2.21	−0.058	0.628
Unobstructed BV		15.45 ± 2.72	0.226	0.056
Ischemia	0.75 ± 0.32			
Obstructed BV		18.74 ± 2.64	−0.310	0.000**
Unobstructed BV		15.48 ± 3.11	0.098	0.270
CRVO				
Non-ischemia	0.74 ± 0.39			
Superior-temporal BV		17.03 ± 1.91	−0.205	0.093
Inferior-temporal BV		18.23 ± 1.97	−0.461	0.000**
Ischemia	0.81 ± 0.25			
Superior-temporal BV		18.47 ± 2.48	−0.259	0.006**
Inferior-temporal BV		19.83 ± 2.30	−0.226	0.017*

BRVO, branch retinal vein occlusion; CRVO, central retinal vein occlusion; FAZ, foveal avascular zone; BV, branch vein. * *p* < 0.05, ** *p* < 0.01.

## 4. Discussion

In patients with RVO, vein occlusion could lead to elevated venous pressure, turbulent blood flow, and overloading drainage capacity, which may cause dilation of retinal veins and capillaries (16). Simultaneously, the intraocular level of VEGF increases sharply during vein occlusion and is the most important mediator responsible for the development of neovascularization and macular edema. Thus, the VEGF inhibitor has been proven to be an effective first-line therapeutic strategy for treating neovascularization and macular edema secondary to RVO (17).

Several researchers hold a view that the VEGF blockade may lead to a poor progression of the retinal nonperfusion area (18, 19), while Campochiaro et al. have reported that the VEGF blockade not only prevented the worsening of retinal ischemia but also promoted the retinal reperfusion (20). The findings of this study were comparable to studies that similarly reported that the FAZ area changed after ranibizumab treatment in patients with RVO. If RVO patients who presented with macular edema already have severe parafoveal capillary dropout before treatment, the macular ischemia may not recover even after the resolution of the macular edema. Moreover, the present study showed that the enlargement of the FAZ area was transient and could return to near baseline at 24 months after injection. Samara et al. measured the FAZ area of healthy subjects (ranging from 0.21 to 0.41mm^2^) by FFA (21). Feucht et al. and Erol et al. reported that enlargement of the FAZ area was observed on FFA images after intravitreal anti-VEGF treatment in patients with diabetic retinopathy or RVO (22, 23). However, other studies have arrived at a controversial conclusion that the FAZ area remained statistically unchanged before and after anti-VEGF therapy (24). Several possible factors contribute to these opposite results, including the follow-up of these studies varied from 6 months to 20 months, the different number of injections, and the patient sample size. The FAZ area of CRVO eyes was larger than that of BRVO eyes due to much greater VEGF activity in the vitreous of CRVO eyes, particularly in the ischemic type.

The present study found the retinal vein diameter of many RVO patients was significantly reduced after anti-VEGF treatment compared with baseline. This phenomenon could be explained by adverse vasoconstrictive effects of anti-VEGF therapy that were reduction of retinal flow velocity and vein diameter (25, 26). In addition, most of the retinal veins in RVO eyes were thicker than those in normal eyes mainly because VEGF could lead to vessel dilation and increased ocular blood flow by enhancing the endothelial nitric oxide synthetic rate and upregulating the activity of VEGF receptors in human endothelial cells (27). In normal eyes, the mean vessel diameters of the superior temporal veins and the inferior temporal veins were shown to be 17.15 ± 1.56 × 10^−2^ mm and 14.86 ± 3.37 × 10^−2^ mm, respectively, indicating that the inferior temporal vein diameter is normally thinner than that the superior temporal vein diameter, which was also proven by Ouyang et al. (28). The present study show that the diameter of superior temporal veins was thinner than that of the inferior ones in eyes with BRVO and CRVO, regardless of whether they were measured at baseline or after treatment. Retinal vein obstruction occurs easily in the superior temporal veins. Weinberg’s study could also partially support the similar result that there was a greater proportion of vein-posterior crossing in superior temporal veins than in inferior temporal veins; thus, most BRVOs occurred in the retinal superior temporal quadrant (29).

As shown in the present study, the FAZ area had a negative correlation with BCVA (LogMAR) after treatment in eyes in both CRVO groups and in the ischemic BRVO group, but not in eyes with non-ischemic BRVO. This result was partially in line with a report from a previous study that an enlargement of the FAZ area correlated negatively with poor visual outcomes (30). However, the correlation of the FAZ area and BCVA was shown differently in many studies. Balaratnasingam et al. demonstrated that the FAZ area measured with FFA was significantly correlated with BCVA in eyes with RVO and diabetic retinopathy, and it was an important predictor for evaluating visual function after treatment of these diseases (14). On the contrary, Remky et al. postulated that the FAZ area was negatively correlated with BCVA in BRVO eyes but not in CRVO eyes (31). There are many reasons for the discrepancy in the relationship between the FAZ area and BCVA in the two types of RVO, which may be due to intraretinal cystic changes, degree of macular edema, disorganization of the retinal inner layers, length and integrity of the ellipsoid zone band. All these factors could affect the improvement of BCVA during follow-up.

In the present study, vein diameter reduced significantly along with the enlargement of the FAZ area after anti-VEGF treatment in eyes with RVO. The obvious vasoconstriction in retinal veins may be interpreted as a return to the normal diameter from a previously vasodilated status due to VEGF inhibition. After anti-VEGF therapy, the diameter of the obstructed branch veins in eyes with ischemic BRVO and the diameter of the branch veins in eyes with CRVO had negative correlations with the FAZ area, but this correlation was not observed in most of eyes with BRVO or the superior temporal branch veins of non-ischemic CRVO eyes. This finding indicated that the branch veins of non-ischemic BRVO eyes and the unobstructed branch veins of ischemic BRVO eyes may still have normal circulation and can maintain their filling degree without depending on the FAZ ischemia status. The obstructed branch veins of ischemic BRVO eyes already have impaired circulation, which can be affected by the adjacent branch retinal veins near the fovea. In the ischemic CRVO groups, the superior and inferior temporal branch veins were both affected by occlusion, and the circulation of inferior ones was weaker than that of superior ones due to anatomical characteristics. Therefore, the diameter of the superior branch veins of non-ischemic CRVO eyes were rarely affected by the FAZ ischemia degree.

## 5. Conclusion

The integration of a FFA platform with image analysis techniques has provided valuable insights into the detection and evaluation of RVO. Consecutive intravitreal ranibizumab injections can cause significant short-term enlargement of the FAZ area and decreased retinal vein diameter in eyes with RVO, although the natural course of RVO may also affect ischemia and visual gain. After 12 months, the FAZ area and retinal vein diameter begin to recover and return to near baseline until 24 months. Ranibizumab therapy may cause macular ischemia to become more severe and affect BCVA for a short time. These findings highlight a potential adverse effect of consecutive ranibizumab therapy, which needs to be taken into consideration in the treatment and prognosis of RVO. The FFA platform with image analysis techniques offers a valuable tool for accurate detection, monitoring, and management of RVO, aiding clinicians in making informed treatment decisions for improved patient outcomes.

## Data availability statement

The original contributions presented in the study are included in the article/supplementary material, further inquiries can be directed to the corresponding authors.

## Ethics statement

The studies involving humans were approved by the Second Affiliated Hospital of Xi’an Jiaotong University of the Medical Ethics Committee. The studies were conducted in accordance with the local legislation and institutional requirements. Written informed consent for participation was not required from the participants or the participants’ legal guardians/next of kin in accordance with the national legislation and institutional requirements.

## Author contributions

DL: Investigation, Methodology, Writing – original draft. ZZ: Formal analysis, Writing – original draft. FW: Data curation, Writing – original draft. BZ: Formal analysis, Writing – original draft. YW: Data curation, Writing – original draft. YZ: Investigation, Writing – review & editing. JL: Investigation, Writing – review & editing.
